# Clinical characteristics and therapeutic evaluation of childhood myasthenia gravis

**DOI:** 10.3892/etm.2015.2256

**Published:** 2015-02-03

**Authors:** ZHI-XIAO YANG, KAI-LI XU, HUI XIONG

**Affiliations:** 1 Department of Neurology, Children’s Hospital of Zhengzhou, Zhengzhou, Henan 450053, P.R. China; 2 Department of Paediatrics, Peking University First Hospital, Beijing 100034, P.R. China

**Keywords:** children, myasthenia gravis, treatment, prognosis

## Abstract

This study aimed to analyze the clinical characteristics, classification and treatment of childhood myasthenia gravis (MG) and address the prognosis through follow-up. The clinical data of 135 children with MG were grouped according to clinical type and therapeutic drugs, retrospectively analyzed and prospectively monitored. Of the 135 MG patients, 85.2% had type I (ocular type), with only 4.2% progressing to systemic MG; 13.4% had type II (general type); and 1.5% had type III (fulminating type). Relapse occurred in 46.1% of the 102 patients that were followed up. The positive rate for the primary acetylcholine receptor antibody was 40.19%, without significant differences among clinical subtypes. The positive rate of the repetitive nerve stimulation frequency test by electromyography was 37.97%. Decreased expression of CD4^+^, CD8^+^, or CD3^+^ was present in 71% of the patients. Thymic hyperplasia was present in 5.93% of the patients, while 1.48% had thymoma. Steroid treatment was effective in the majority of the patients. Ocular type MG was common in this cohort of patients. The incidence and mortality of myasthenia crisis were low, the presence of concurrent thymoma was rare and only a limited number of children developed neurological sequelae.

## Introduction

Myasthenia gravis (MG) is an autoimmune disease primarily mediated by the anti-acetylcholine receptor antibody (AchR-Ab), which is associated with a variety of immune molecules and occurs in neuromuscular junctions (1,2). MG is characterized by skeletal muscle weakness, in parts of or the entire body following continuous repetitive motion, fatigability and fluctuating levels of muscle weakness, as well as other symptoms, including diplopia, ptosis and difficulties in mastication and swallowing. It was previously demonstrated that genetic factors play an important role in the pathogenesis of MG (3). In addition, viral or bacterial infections, such as poliovirus and *Escherichia coli*, may be involved in the pathogenesis of MG (4,5). However, there is no evidence indicating a definitive MG pathogenesis. Anti-cholinesterase drugs, non-specific immunosuppressive agents, thymectomy and plasmapheresis are currently the main treatment options (6–8). MG is more common among Chinese children, who exhibit significantly different clinical characteristics compared to those observed in adults. The aim of this study was to analyze the clinical characteristics, classification and treatment of MG in children and understand the prognosis through follow-up, in order to determine the optimal treatment options and improve the patients’ quality of life. In addition, we present the conclusions drawn from the age-related clinical characteristics, treatment and prognosis.

## Materials and methods

### Experimental subjects

A total of 135 children diagnosed with MG, who were admitted to the Children’s Hospital of Zhengzhou between January, 2008 and January, 2014, were included in this study. The patients included 57 males and 78 females. The age at onset ranged between 4 months and 15 years, with an average age at onset of 3 years and 2 months. Three patients were aged <1 year, 65 were aged 4 months-3 years, 41 were aged 4–7 years, 20 were aged 8–12 years and 6 patients were aged 13–15 years. The age at the time of the doctor’s visit ranged between 4 months and 15 years. A total of 81 patients were newly diagnosed and 54 received irregular treatment or experienced recurrence following treatment.

This study was conducted in accordance with the principles of the Declaration of Helsinki and received approval from the Ethics Committee of the Peking University First Hospital. Written informed consent was obtained from all the participants or their legal custodians.

### Diagnostic criteria and classification

The following clinical symptoms were considered indicative of MG: muscle fatigue; muscle weakness mitigated in the morning and worsening in the evening; and progressive muscle weakness following continuous or repeated contractions, mitigated with rest or cholinesterase inhibitor use.

The diagnosis is confirmed with a positive result on one of the following tests (9): fatigue test or sulfuric acid neostigmine test (every 0.025–0.05 mg/kg, no improved muscle weakness after 15–30 min, restitution after 1.5 h); repetitive nerve stimulation frequency test (low-frequency repetitive stimulation of muscle action potential amplitude, decreasing attenuation >10%); or positive determination of serum anti-AchR antibody.

The MG classification was determined using the modified Osserman method as follows: type I (ocular type MG; OMG) with only extraocular muscle involvement, including type Ia (ptosis) and type Ib (ptosis and eye fixation); type II (general type MG; GMG) including type IIa (eye and limb muscle weakness) and type IIb (general weakness during mastication or swallowing and dysarthria); type III (fulminating type) with sudden weakness and myasthenic crisis; type IV (flaccid type), which progresses from type I or II and peaks at 2–3 years; and type V (amyotrophic type) (4).

### Methods

The clinical characteristics, affiliated examinations, treatment and prognosis of the patients were retrospectively analyzed and prospectively followed up.

### Statistical analysis

Data analysis was performed using SPSS 14.0 software (SPSS Inc., Chicago, IL, USA). Continuous data are expressed as means ± standard deviation or minimum and maximum. Enumeration data are expressed as the number of patients and frequency. Groups based on the enumeration data are expressed using the number of patients and percentage of patients within each group. The comparisons among the enumeration data groups were performed using Chi-square tests or Fisher’s exact tests. *P*<0.05 was considered to indicate a statistically significant difference.

## Results

### Clinical manifestations and classification

The treatment duration was <1 month in 55 patients, 1–3 months in 29 patients, 4–6 months in 12 patients, 7–12 months in 7 patients and 1–13 years in 32 patients. The majority of the patients with a disease course of ≤6 months were newly diagnosed patients, while the majority of the patients with a disease course of >6 months received irregular treatment or experienced relapse following treatment. All the patients experienced manifestations involving clinical muscle weakness during hospitalization: only 61 patients (45.2%) had ptosis; 54 patients (40.0%) had a combination of ptosis and eye movement disorders; 14 patients had ocular symptoms with associated limb weakness; 4 patients had a combination of bulbar muscle involvement, such as facial, tongue, throat and mastication muscle weakness; and 2 patients had respiratory muscle involvement. Type I was present in 115 patients (85.2%), type II in 18 patients, type III in 2 patients (1.5%) and none of the patients had type IV or V (Table I).

A 12-year-old female patient developed hyperthyroidism following multiple disease recurrences. A 2.3-year-old female patient also had selective IgA deficiency. A total of 6 patients experienced a myasthenic crisis during the course of the disease, including 4 patients who underwent ventilator-assisted respiratory therapy and 2 patients who succumbed to severe pulmonary infection. An 18-year-old patient was misdiagnosed with muscular dystrophy; 4 patients were misdiagnosed with oculomotor nerve palsy, including 1 patient that was misdiagnosed for 4 years; 5 patients were misdiagnosed with Guillain-Barré syndrome; and 1 patient was misdiagnosed with idiopathic epilepsy. Two patients only exhibited limb-girdle muscle weakness, with an early misdiagnosis of psychogenic illness; and 2 patients were misdiagnosed with brain stem encephalitis. These patients were ultimately diagnosed using electromyography (EMG), serum AchR-Ab detection and response to treatment.

### Serum antibody

Serum AchR-Ab (Beijing-Hui Bogosian Biotechnology Co., Ltd. Beijing, China), anti-ryanodine receptor calcium release channel antibody (RyR-Ab) (Shanghaikamaishu Biotechnology Co., Ltd., USA) and anti-catenin antibody (Titin-Ab) (Shanghai Chemical Reagent Co., Ltd., Shanghai, China) were detected by ELISA and monitored during follow-up. Serum AchR-Ab was positive in 43 (40.19%) of the 107 primarily diagnosed patients and the positivity rate was not significantly different among the clinical subtypes. Of the 28 primarily diagnosed patients in whom serum RyR-Ab was detected, 3 patients (10.71%) were also positive for AchR-Ab Serum Titin-Ab was detected in 30 patients and 12 patients (40%) tested positive for both Titin-Ab and AchR-Ab. Computed tomography (CT) did not reveal thymoma in 9 of these patients and chest radiography was normal in 3 patients who did not undergo chest CT. Of the 18 patients with negative Titin-Ab, 7 were positive for AchR-Ab, whereas the remaining 11 patients were negative. Following treatment for 6–24 months, 24 patients underwent dynamic follow-up of serum AchR-Ab and 9 of the 17 patients (53%) who were initially negative for AchR-Ab were found to be positive; in 2 of these patients, AchR-Ab remained positive from the end of treatment to clinical recovery during the follow-up. Of the 7 patients with positive AchR-Ab, 4 had negative AchR-Ab with symptomatic remission.

### EMG

EMG (Model ZET-100; Shanghai Zhong Ren Electronic Instrument Co., Ltd.) was conducted in 79 patients, with routine inspection of the facial, ulnar, median and common peroneal nerves; the skin over the muscles was routinely disinfected and the needle poles were quickly inserted. The pin position was selected as the center of the abdominal muscle or movement points and a 1 or 2 needle electrode was inserted into every muscle. The different (upper, middle, or lower) muscle insertion sites were selected when the parameters of the motor unit potential were determined. The repetitive nerve stimulation frequency test of the 30 patients (37.97%) exhibited attenuation, whereas none of the ocular patients exhibited peripheral nerve attenuation.

### Immunological parameters

As regards the 63 patients who underwent immunoglobulin and the 101 who underwent CD series tests, the IgG, IgA and IgM results were all normal, 1 patient exhibited decreased IgA and 71% of the patients had CD series count abnormalities, with the majority expressing decreased CD4^+^ and CD8^+^ and some patients expressing increased CD19^+^ (Table I).

### Imaging

Routine radiographic examination of the anteroposterior chest was performed in 131 patients and 57 patients underwent simultaneous single-slice spiral CT of the thymus; 8 patients (6.11%) exhibited thymic hyperplasia and 2 patients (1.5%) had thymoma that was pathologically diagnosed, without Titin-Ab detection.

### Other tests

Following blood biochemistry, muscle enzyme and thyroid function tests, 1 patient had hyperthyroidism with positive thyroglobulin and anti-thyroid peroxidase antibodies. The echocardiography results of all the patients were normal.

### Treatment

Oral pyridinium bromide neostigmine (5–7 mg/kg/day, 3 times/day) was administered to 39 patients for 6 months to 1 year, depending on the condition. Oral prednisone was administered to 26 patients, with transitional reduction: the initial 1.5–2 mg/kg/day was gradually reduced to 7.5–10 mg every morning and with optimal symptom control (4–6 weeks later), the therapy was maintained for 1–2 years. Seven patients were treated with pyridinium bromide neostigmine combined with prednisone that was transitionally reduced; for the 61 patients treated with methylprednisolone pulse therapy at a dose of 15–20 mg/kg/day, each week-long course consisted of a continuous administration for 3 days and no treatment for 4 days for 1–3 courses, followed by 1.5–2 mg/kg/day oral prednisone, which was reduced after 4–6 weeks and followed by a reduction of 0.5 mg/kg oral prednisolone every two weeks in transitional decrements. After it was decreased to 0.5 mg/kg/day it was maintained at that concentration for a period of 1–2 years. Two patients were routinely administered prednisone for 1–2 years postoperatively following thymectomy. In addition, 10 patients were treated with 400 mg/kg/day intravenous gamma globulin (IVIG) due to repeated recurrence of the disease, once a day for 5 days. A total of 9 patients received cyclosporin A, azathioprine and other immunosuppressive agents. The duration of improved muscle weakness, duration of maximum effect and recurrence (e.g., symptoms reappearing following discontinuation of the drug) were monitored during the follow-up for inpatients and outpatients.

Regarding the four types of treatment (based on the initial treatment program), 39 patients were classified to the single pyridinium bromide neostigmine group, 26 patients to the transitional reduction of oral prednisone group, 7 patients to the pyridinium bromide neostigmine in combination with reduction of prednisone group and 61 patients to the methylprednisolone pulse therapy group. The mean treatment onset time was 13 (10–17), 11 (4–15), 8 (1–12) and 2 (1–6) days, respectively. The mean duration of the maximum effect was 74 (23–212), 52 (19–174), 48 (7–125) and 18 (3–54) days, respectively, with no significant differences among the subtypes. The follow-up of the treatment lasted for 2–15 years (until January, 2010), during which time 29 patients were lost to follow-up, with the remaining 106 MG patients completing the follow-up. During the treatment, relapse occurred 1–2 times in 43 patients, 3–5 times in 10 patients and >5 times in 1 patient; the onset of relapse occurred within 2 years for 84.6% of the patients. The frequency of relapse was not significantly different between the single pyridinium bromide neostigmine and the steroid groups, but the lower rate of recurrence in the steroid treatment group was statistically significant (Table II). In the 10 patients who were administered IVIG due to repeated recurrence, their condition improved markedly in the first 10–15 days following the injection.

Due to frequent relapses or steroid dependence/resistance, 2 patients received additional azathioprine and 1 patient was switched to cyclosporin A treatment due to bone marrow suppression. A total of 9 patients were administered cyclosporin A and 1 of these patients was switched to sulfur azathioprine due to suboptimal efficacy.

### Adverse reactions

All the early adverse reactions that developed in patients who underwent steroid therapy alone involved different degrees of Cushing’s syndrome, weight gain, hypertension (n=1) and weakness exacerbation (n=2). Patients who were administered pyridinium bromide neostigmine combined with steroid therapy exhibited no early exacerbation of weakness. Upon symptom improvement, the steroid dose was reduced. During the maintenance period, the majority of the patients exhibited no other long-term adverse effects, such as severe infections, fractures, abscess formation or gastrointestinal hemorrhage.

### Follow-up

The longest follow-up period was 15 years in 1 patient, who is currently 27 years old, without symptoms of muscle weakness. A total of 4 patients experienced sequelae in the form of ocular movement disorders and 2 patients succumbed to the disease.

## Discussion

MG is an acquired autoimmune neuromuscular transmission disorder. The pathophysiology involves the production of AchR-Ab and the inhibition of AchR, reducing the number of functional AchR in the neuromuscular junction, which leads to an abnormal decline in the motor endplate membrane potential. Studies conducted in Europe and the United States have reported a prevalence of 14–1.5/1.0×10^5^, with an uncommon MG onset during childhood (10–15%) (10). However, a recent study conducted in China indicated that the onset of MG in children is not rare in China (11), with similar rates to those in Japan (12), indicating that pediatric MG is relatively common in Asia. The results of the present study are consistent with those available in the literature and demonstrated a relatively early age at onset (50.3% of the patients were aged <3 years), with a marginally higher rate among females (56.3%), common relapse (50.9%) and a higher relapse rate within the first 2 years. A higher number of children with MG in European countries progress to GMG within 2 years. Research in Japan has indicated that 75% of patients with OMG had recessive GMG (13), i.e., no clinical symptoms were present, but EMG detected repetitive nerve stimulation frequency attenuation in other nerves in addition to the facial nerve in patients who were diagnosed with latent GMG. Our patients rarely progressed to GMG and EMG did not suggest an attenuation phenomenon in other neurological examinations of children with OMG (rare recessive GMG). However, detailed history, increased number of fatigue tests and EMG sites and long-term follow-up of the patients should be performed in order to increase the accuracy of typing.

Spalek *et al* (14) investigated 768 adult MG patients and found that 112 patients (14.15%) also suffered from other autoimmune diseases. In the present study, one 12-year-old female patient experienced repeated recurrence of bulging eyes over the first 3 years following treatment. The initial examination for AchR-Ab was negative, but was positive at recurrence; testing for antithyroid antibodies was also positive. Graves’ disease is reportedly rare in patients with MG (0.2%), whereas 5–10% of patients with MG have autoimmune thyroid diseases (15). Antithyroid antibodies are more common in OMG and reportedly more common in Asian patients (16). In the patients in the present study, concurrent Graves’ ophthalmopathy was not as common. It is difficult to determine when proptosis and ptosis appear, but serum antibody tests may help with the diagnosis, particularly in children with atypical clinical manifestations and no definitive diagnosis. Therefore, routine testing for antithyroid antibodies and thyroid function should be performed and other autoimmune diseases should be considered.

Of the patients who were initially AchR-Ab-negative, 53% became positive during treatment or following relapse, which may be a reaction during a different period of the same disease or may be due to differences in individual immunity and associated with the level of antibody titers and differences in secretion in various tissues and organs (17). Dynamic follow-up may increase the antibody positivity rate in children with MG; however, as a causative factor, AchR-Ab was long-standing in certain patients, indicating that long-term treatment may lower the risk of recurrence.

It was previously demonstrated that, in addition to being AchR-Ab mediated, MG pathogenesis also involves other antibodies or molecules, such as Titin-Ab, which were recently found to be highly relevant with thymoma-related MG (MGT). The Titin-Ab positivity rate in MGT was as high as 80–90%, which is of high diagnostic value for MGT. The level of serum Titin-Ab was also found to be significantly correlated with MGT severity and AchR-Ab levels. The detected positivity rate of Titin-Ab in the present group was 40% and the positive patients were also positive for AchR-Ab, although the imaging studies revealed no evidence of thymoma. However, follow-up of the Titin-Ab-positive patients was necessary, particularly for diagnosis by enhanced CT. Foreign studies have reported that positive RyR-Ab is associated with GMG, particularly with severe oropharyngeal muscle weakness that often occurs during myasthenic crises (12); however, no such correlation was found in the 3 positive patients who were followed up. In this group, 71% of patients had abnormal CD series, with the majority displaying decreased CD4^+^ and CD8^+^, indicating that immune cells also play a role in the pathogenesis of MG.

MG is usually mitigated in the morning and worsens in the evening and patients may experience incomplete paralysis in the later stages. Two patients with upper eyelid, limb and bulbar muscle involvement experienced sustained myasthenia. Fatigue and drug testing was negative and the patients were diagnosed with brain stem encephalitis. Other patients were misdiagnosed with oculomotor nerve palsy, muscular dystrophy, Guillain-Barré syndrome, mitochondrial diseases, cardiomyopathy and other symptoms. These patients were all diagnosed by positive AchR-Ab and EMG and recovered during the follow-up after treatment; therefore, several differential diagnoses should be considered in the clinical setting. Early onset should also be distinguished from congenital myasthenic syndrome. Another 9 patients were had negative neostigmine test results, but the AchR-Ab were present or the results of the EMG were positive, and steroid treatment was effective. Therefore, acquired autoimmune MG should also be considered.

Although the pathogenesis of MG is clear, the currently available treatments are not immune- or antigen-specific. The international and domestic treatment standards are currently not uniform, including hormone measurements, course of treatment and selection of second-line drugs. Acetylcholinesterase inhibitors were suitable for all MG patients, except those undergoing cholinergic crisis, and may significantly reduce muscle weakness and impede AchR repair; therefore, they should not be used as a single agent in the long term. Many advocate the use of steroid therapy in the early stages (18) to prevent transiently increased weakness. Corticosteroids, which are the most commonly used first-line immunomodulatory therapy for MG, exert multiple effects on T-cell, B-cell and macrophage function. Kupersmith (18) reported that prednisone may delay and reduce the incidence of GMG after >4 years of follow-up and effectively control recurrence.

The following advantages of methylprednisolone pulse therapy were observed: i) satisfactory effect in the short term, with rapidly improving symptoms; ii) ability to reduce or discontinue cholinesterase inhibitor use in the short term may simplify treatment; iii) relatively few adverse reactions, effectively controlled during hospitalization; and iv) ability to return to normal routine as soon as possible and continue with outpatient observation and treatment. However, a retrospective analysis regarding MG crisis indicated that it may be associated with inappropriate methylprednisolone pulse therapy (19). Our data demonstrated that the average time to onset and duration of the maximum effect in the steroid pulse group was superior to the transitional reduction of the oral prednisone group and the pyridinium bromide neostigmine in combination with reduction of prednisone group. Except for the two MG patients who received steroids alone, the remaining children did not experience MG exacerbation or crisis. Steroid treatment of short duration resulted in early relapse; a treatment duration of ≥2 years was considered more appropriate. In addition to Cushing’s facies, weight gain during the early stages of medication and high blood pressure occurred in 1 patient, but there were no severe adverse drug reactions in the majority of the patients who were administered steroid therapy. However, regular referrals to ophthalmology, blood glucose monitoring, blood pressure monitoring, a low-salt diet, use of cod liver oil supplements and monitoring of calcium and bone density are recommended. If the illness recurs during treatment, the steroid dose may be restored to pre-reduction levels; alternatively, cholinesterase inhibitors, IVIG, or other immunosuppressive agents may be added in the short-term if the manifestations are severe.

Azathioprine, which is the most common immunosuppressant used long-term to treat MG in addition to corticosteroids, was proven to be effective through randomized controlled trials. Cyclosporine A is the only treatment confirmed to be effective in improving clinical symptoms and decreasing anti-AchR serum levels compared to a control group in a randomized, double-blind, controlled trial. In the Children’s Hospital of Zhengzhou, cyclosporine A treatment was used for 1.5 years in children with repeated relapses with steroid-resistant or -dependent disease, on the basis of observed severe side effects with azathioprine, reports on the effectiveness of cyclosporine A treatment (20) in addition to our own experience with cyclosporine A and the lack of severe adverse events with cyclosporine A treatment. Other clinically applied drugs and treatment methods, such as cyclophosphamide, mycophenolate mofetil, tacrolimus, plasmapheresis and thymectomy were also used. Recently, rituximab and antigen-specific plasma exchange were also used, but more experience is required. Thymectomy in pediatrics should be cautiously performed. In addition, the use of drugs that aggravate muscle weakness, such as aminoglycoside and macrolide antibiotics, should be avoided; MG exacerbation may occur during surgery (21). When the non-ionic contrast iohexol is used in enhanced CT, no aggravated weakness is observed.

In summary, MG in children is more common in China. OGM is common and there are fewer patients who progressed to GMG, fewer patients with concurrent hyperthyroidism and a low incidence of thymoma. The overall prognosis is good, with fewer neurological sequelae, although visual impairment should also be considered. The significance of integrated management, long-term follow-up, standardized treatment, education for children and parents and psychological support should be emphasized.

## Figures and Tables

**Table I:** Clinical information of 135 cases of patients.

Characteristics	Patient no. (n=135)	%
Gender		
Male	57–59	40.0–43.7
Female	78–76	60.0–46.3
Upper respiratory infection before onset	36	26.7
Age at onset (5 months–15 years)		
5 months-3 years	68	50.4
4–7 years	41	30.4
8–12 years	20	14.8
13–15 years	6	4.4
Clinical classification at onset (Osserman type)		
Ia	61	45.2
Ib	54	40.0
IIa	14	10.3
IIb	4	3.0
III	2	1.5
Relapsing cases (times)	54/106^a^	50.9
1–2	43/54	79.6
3–5	10/54	18.5
>5	1/54	1.9
Auxiliary examinations		
AchR-Ab-positive	43/107^b^	40.2
RyR-Ab-positive	3/28^c^	10.7
Titin-Ab-positive	12/30^d^	40.0
Negative Ab turned positive during follow-up	9/17^e^	53.0
Abnormal RNS	30/79^f^	38.0
Abnormal thyroid function	17/86^g^	26.1
Abnormal immunoglobulin	1/63^h^	1.6
Abnormal CD series	71/101^i^	71.0
Thymic hyperplasia, thymoma	2/131^j^	1.5

a A total of 29 patients were lost to follow-up.

b Primarily diagnosed patients.

c Number of patients who underwent the RYR-Ab test.

d Number of patients who underwent the Titin-Ab test.

e Patients initially negative for AchR-Ab.

f,g,h,i Number of patients who underwent the respective tests.

j Patients who underwent radiographic examination of the chest.

AchR-Ab, anti-acetylcholine receptor antibody; RYR-Ab, anti-ryanodine receptor calcium release channel antibody.

**Table II:** Therapeutic and follow-up information of the patients (n=106).

Therapeutic schemes	Total no.	Cured, no. (%)	Relapse (no.)
Ocular type^a^	88	43	45
Pyridostigmine group	21	7 (33)	14
Steroid group	67	36 (54)	31
General type^b^	18	9	9
Pyridostigmine group	6	4 (67)	2
Steroid group	12	5 (42)	7
Course of treatment (years)			
<1	30	10 (33)	20
1–2	74	40 (54)	34
>2	2	2 (100)	0

a χ^2^=2.662, P=0.103.

b Exact probability test, P=0.620.
